# Beta-Blockers Reduced the Risk of Mortality and Exacerbation in Patients with COPD: A Meta-Analysis of Observational Studies

**DOI:** 10.1371/journal.pone.0113048

**Published:** 2014-11-26

**Authors:** Qingxia Du, Yongchang Sun, Ning Ding, Lijin Lu, Ying Chen

**Affiliations:** Department of Respiratory Medicine and Department of Emergency, Beijing Tongren Hospital, Capital Medical University, Beijing, 100730, China; University of Dundee, United Kingdom

## Abstract

**Background:**

Cardiovascular disease is a primary cause of death in patients with chronic obstructive pulmonary disease (COPD). Beta-blockers have been proved to reduce morbidity and improve survival in patients with cardiac diseases. But the effects of beta-blockers on outcomes in patients with COPD remain controversial. The objective of this meta-analysis was to assess the effect of beta-blockers on mortality and exacerbation in patients with COPD.

**Methods:**

An extensive search of the EMBASE, MEDLINE and Cochrane was performed to retrieve the studies of beta-blockers treatment in patients with COPD. The random effects model meta-analysis was used to evaluate effect on overall mortality and exacerbation of COPD.

**Results:**

Fifteen original observational cohort studies with a follow-up time from 1 to 7.2 years were included. The results revealed that beta-blockers treatment significantly decreased the risk of overall mortality and exacerbation of COPD. The relative risk (RR) for overall mortality was 0.72 (0.63 to 0.83), and for exacerbation of COPD was 0.63 (0.57 to 0.71). In subgroup analysis of COPD patients with coronary heart disease or heart failure, the risk for overall mortality was 0.64 (0.54–0.76) and 0.74 (0.58–0.93), respectively.

**Conclusion:**

The findings of this meta-analysis confirmed that beta-blocker use in patients with COPD may not only decrease the risk of overall mortality but also reduce the risk of exacerbation of COPD. Beta-blocker prescription for cardiovascular diseases needs to improve in COPD patients.

## Introduction

Chronic obstructive pulmonary disease (COPD) is a growing concern in public health and one of the leading causes of death worldwide [1]. Many patients with COPD also have concomitant cardiovascular diseases, and about 37% of patients with COPD die from coronary heart disease or heart failure, compared with 34% of those die from COPD itself [2]. Intriguingly, impaired lung function seems to be an independent and powerful risk factor for arrhythmias, coronary events, and a specific predictor of cardiac mortality [3]. These coexisting conditions present therapeutic challenges for clinicians.

The beta-adrenergic system contains β_1_ and β_2_ receptors that coexist in varying concentrations in the lung, heart, and peripheral tissues. The β_2_ receptors are found predominately in bronchial and vascular smooth muscles, adrenergic nerves, and peripheral leukocytes. The β_2_-agonists, such as salmeterol and albuterol, are widely used as bronchodilators in the treatment of COPD and asthma. Beta-blockers exert the reverse physiologic effects of β_2_-agonists and may be expected to have deleterious respiratory effects.

In clinical studies, the use of beta-blockers has been demonstrated to reduce morbidity and mortality in patients with coronary heart disease or heart failure [4],[5],[6]. However, practice guidelines usually list COPD and asthma as contraindications to beta-blocker use, because the presumed competition with β_2_-agonists and bronchoconstrictive properties of beta-blockers [7]–[15]. Physicians are often reluctant to prescribe beta-blockers in patients with COPD for fear of inducing adverse reactions and bronchospasm [16]. Conversely, there is increasing evidence that beta-blockers could temper the sympathetic nervous system and theoretically exert beneficial effects in patients with COPD by reducing heart rate and prolonging the diastolic period of cardiac cycle and thus reducing the ischemic burden [17]. Other studies have shown that beta-blockers use in patients with COPD is associated with a significant reduction in COPD exacerbations and COPD mortality [26]. This meant that patients with COPD may miss out on the benefits of beta-blockers, especially those post-myocardial infarction and heart failure. In one Cochrane review of 19 randomized, blinded, placebo-controlled studies on single-dose treatment and 10 on continued treatment of cardioselective beta-blockers in patients with asthma or COPD, beta-blockers reduced FEV1 by 7.46% (5.59%–9.32%) and showed no significant harm [19]. The authors concluded that beta-blockers should not be routinely withheld from patients with COPD and concurrent heart failure and/or coronary artery disease. But the selected trials, however, were short in duration and did not include those with moderate-severe reactive airway disease and elderly patients [19].

The objective of this analysis was to evaluate the benefits of beta-blocker use on mortality and exacerbation in patients with COPD.

## Methods

This meta-analysis was carried out and reported according to the Preferred Reporting Items for Systematic Reviews (PRISMA) [20].

### Literature Search

A comprehensive search of the EMBASE, MEDLINE and Cochrane databases was performed to identify all relevant human clinical trials on beta-blockers use in patients with COPD, published between 1966 and June 2013. Terms used in the search were: 1) Beta-blockers, adrenergic antagonist, sympatholytic or adrenergic receptor blocker. 2) Obstructive lung disease, obstructive airway disease, obstructive pulmonary disease, COPD. Only studies involving human subjects were included. Titles for relevance from this search were reviewed, and all subject heading and abstracts were examined. The search was further augmented by reviews and scanning references of retrieved studies. Literature search was not limited by languages of the published papers.

### Inclusion Criteria

Studies were included if they (1) Studied population originating from a well-established general cohort; (2) Clearly defined beta-blockers use as either a primary exposure in the study or used in a subgroup of patients with COPD,with clearly defined exacerbation; (3) Presented relative risks or odds ratios for mortality or exacerbation and their corresponding confidence intervals or gave enough data to calculate these parameters; (4) Had a follow-up duration of at least 6 months. Exacerbation included hospital admission for an exacerbation of COPD, oral corticosteroid use, or a pulsed-dose prescription of prednisolone.

### Data extraction

Two investigators independently extracted data from the selected articles, and the results of two reviewers were compared, and differences were resolved by discussion and consensus. The first author’s surname and the year of publication of the articles were used for the identification purpose. Study characteristics included in the data extraction form were as follows: authors’ names, publication year, study design, sample size, study population, age (mean or range), outcome of the interest (mortality reduction), type of risk factors, adjusted odds ratio or relative risks (RR) and 95% confidence intervals (CI), covariates adjusted for. Only published data from the studies were included in the analysis.

### Methodological Quality

Newcastle-Ottawa Scale tool (available at: http://www.ohri.ca/programs/clinical_epidemiology/oxford.asp) was used to evaluate the quality of the cohort studies.

### Statistical Analysis

The RRs for overall mortality and exacerbation in each study were pooled using the random-effects model for dichotomous outcomes. The data were analyzed separately for mortality and exacerbation. The random-effects model was chosen because heterogeneity was noted in the analysis.

In order to evaluate the effect of beta-blockers treatment in COPD patients with heart failure or coronary artery disease, we performed a subgroup analysis to evaluate the treatment response for those participants known to have these comorbid conditions. In five of the studies, all COPD participants had comorbid coronary artery disease conditions such as angina pectoris, after coronary artery bypass grafting, myocardial infarction. The second subgroup was patients who had COPD with heart failure. In the other studies, the comorbidities in COPD participants were not specified. The Begg test was used to assess the publication bias [21]. Statistical heterogeneity between studies was examined with Cochran’s Q test and reported as I^2^ value [22]. Sensitivity analysis was used to evaluate the influence of individual studies on the summary effect by looking at the individual influence of the study and then repeating the analysis by excluding the study with the largest weights.

All analyses were done using Stata Software (version 12.0 Stata Corporation, College Station, TX, USA). Statistical significance was taken as two sided p<0.05.

## Results

### Search Results

**Figure 1:**
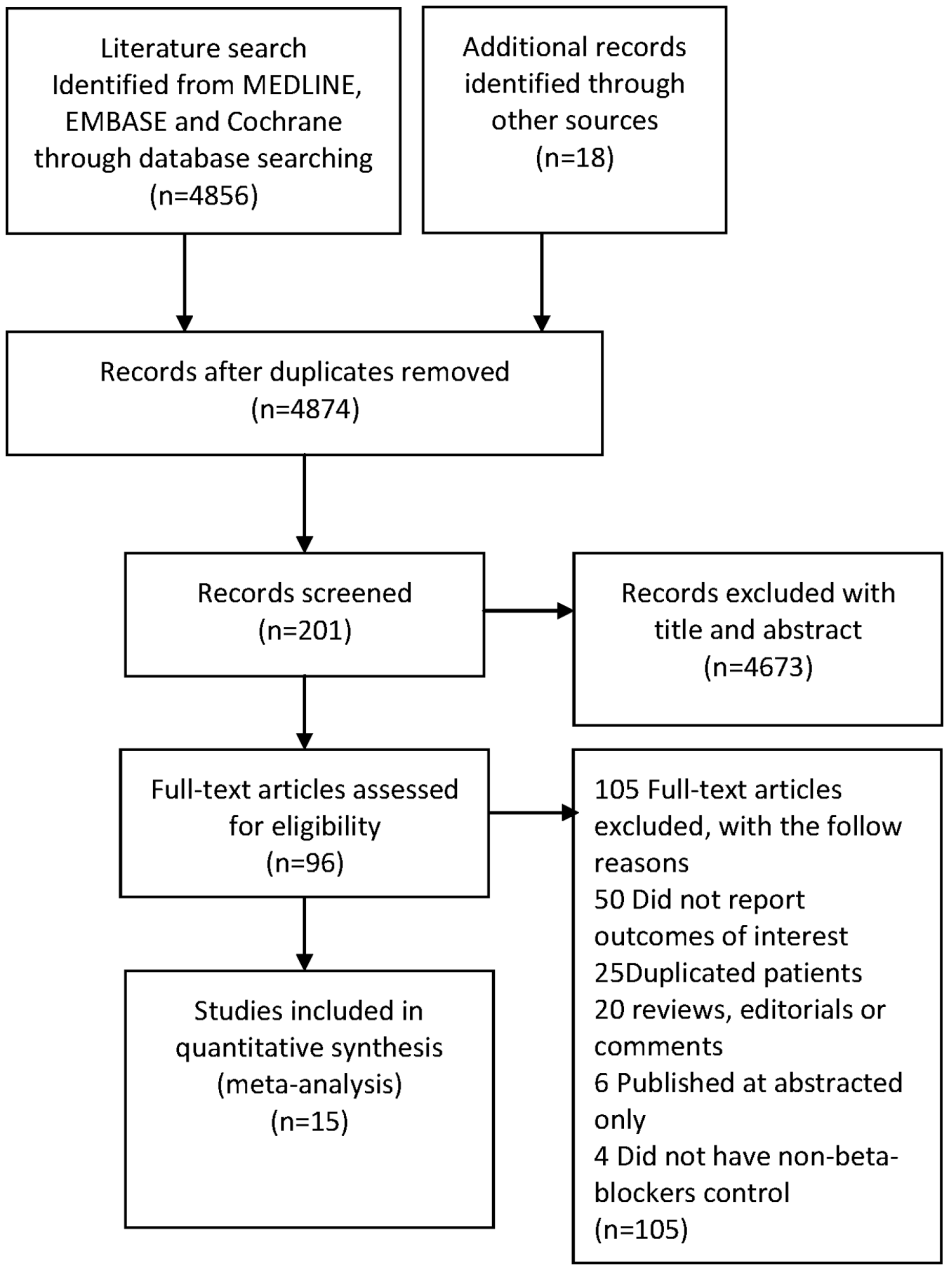
Study flow diagram in this meta-analysis.

The initial search yielded 4,856 unique titles and abstracts from MEDLINE and EMBASE databases and approximately 201 potentially relevant articles identified. After review of articles and bibliographies, 96 articles of beta-blockers in patients with COPD were found. Of these articles, 15 cohort studies fulfilled our inclusion criteria. The details of study selection flow diagram were explicitly described in Fig. 1.

### Study Characteristics

The characteristics of each study were shown in Table 1. Of these studies, 5 studies looked at subjects with COPD who also had coronary heart disease [23],[24],[30],[31],[34]. Three studies looked at chronic heart failure in COPD patients [25],[27],[36]. There were a total of 121,956 participants with ages ranging from 45 to 80 years and mostly older than 60 in these studies. The studies had treatment duration from 1 year to 7.2 years. The effect estimations of relative ratios (RRs) of exacerbation were provided in 6 studies. The quality of the included OSs was displayed in Table S1 (median score, 8.4; range, 8 to 9, assessed by Newcastle-Ottawa Scale tool).

**Table 1:** Characteristics of Studies Included in the Meta-analysis.

Author/year	Design/duration	Participa-ntsNo.	Population	relative risk(95% CI)	Covariates
Gottlieb/1998 [23]	Retrospective cohort/two years	41814	age 73.3±8.8, COPD subjects with previous history of myocardial infarction	Mortality (0.60, 0.57–0.63)	Unadjusted
Chen/2001 [24]	Retrospective cohort/one year	10988	age<65, COPD or asthma subjects with previous history myocardial infarction	Mortality (0.86, 0.73–1.00)	Age, sex, comorbidities, CAD, cardiovascular drugs, physician speciality
Sin/2002 [25]	Retrospective cohort/median 21 months	3834	age 79±8, COPD subjects with heart failure	Mortality (0.78, 0.63–0.95)	Age, sex, comorbidities, cardiovasculardrugs, physician speciality
taszewsky/2007 [27]	Retrospective cohort/median 23 months	628	age 67±9, COPD subjects with heart failure	Mortality (0.55, 0.37–0.82)	Unadjusted
Au/2004 [26]	Retrospective cohort/two years	1966	age 68.1±9.9, Veteran Affairs COPD subjects with hypertension	Mortality (0.56, 0.16–1.93);ECOPD (0.65, 0.29–1.48)	Comorbidity, age, history of COPD, bronchodilators, smoking, coronary artery disease, diabetes
Dransfield/2007 [29]	Retrospective cohort/−	825	age 68±11, COPD subjects with acute Exacerbation	Mortality (0.39, 0.14–0.99);ECOPD (0.46, 0.21–1.04)	Age, CHD, CHF, liver disease, COPD exacerbations, malignancy, smoking, FEV1
Van Gestel/2008 [28]	Retrospective cohort/median 5 years	1205	COPD subjects with vascular disease	Mortality (0.73, 0.60–0.88)	Age, sex, hypertension, hypercho- lesterolemia, diabetes, renal func- tion, smoking, BMI, CAD, FEV1, cardiovascular drugs
Hawkins/2009 [30]	Retrospective cohort/median 25 months	1258	age 68.1±9.9, COPD subjectswith myocardial infarction	Mortality (0.74, 0.68–0.80)	Unadjusted
Olenchock/2009 [31]	Retrospective cohort/two years	12967	COPD or asthma subjects withacute coronary syn- dromes	Mortality (0.52,0.45–0.60).	Unadjusted
Rutten/2010 [32]	Retrospective cohort/7.2 years	2230	Age 64.8±11.2, COPD primary care	Mortality(0.68,0.56–0.83) for Cardio-selective BB,(0.82,0.61–1.10) fornon- selective BB; ECOPD(0.71, 0.60–0.83).	Age, sex, diabetes, hypertension, CAD, CVD drugs, pulmonary drugs
Short/2011 [33]	Retrospective cohort/mean 4.35 years	5977	Mean age 69.1 years, COPD primary care	Mortality(0.78,0.67–0.92);ECOPD (0.39, 0.32–0.48).	CAD and Respiratory disease, age, sex, diabetes, smoking, FEV1, cardiovascular drugs
tefan/2012 [35]	Retrospective cohort/two years	35082	Age 73(65–80), COPD with heart failure, hypertension, ischemic heart disease	In hospital mortality (0.88, 0.71–1.09); ECOPD(0.98, 0.77–1.24).	Age, sex, underlying cardiovascular condition, ischaemic heart disease, congestive heart failure, drug, et al.
Ekström/2013 [37]	Prospective cohort/4 years	2249	age>49, Severe COPD	mortality (1.19, 1.04–1.37).	Age, sex, body mass index, world heath organization performance status, resting blood gas tensions breathing air,comorbidities, and concomitant medication
geloni/2013 [34]	Retrospective cohort/median 36 months	208	COPD after coronary artery bypass grafting	Mortality (0.38, 0.20–0.71);ECOPD (1.02, 0.046–22.5).	Unadjusted
Mentz/2013 [36]	Retrospective cohort/60–90 days	725	age 73(63–80), COPD with heart failure	Mortality (0.89, 0.59–1.35)	Unadjusted

### Quantitative Data Synthesis

**Figure 2:**
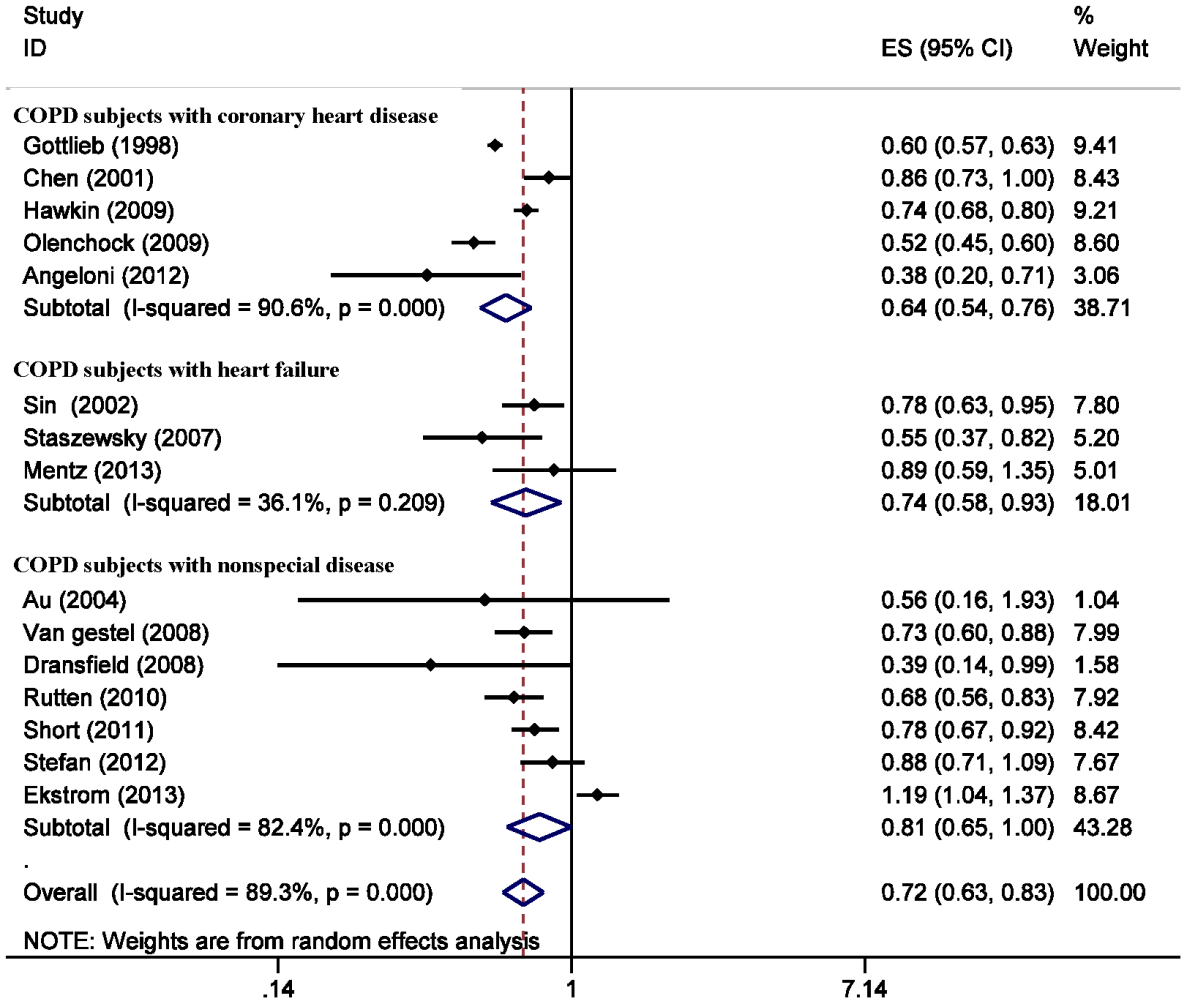
Forest plot showing beta-blockers use and mortality risk in COPD patients. Three subgroups were analysis according to the comorbid conditions of COPD. This Forest plot represents the relative risk (RR) (95% confidence interval) for mortality in COPD patients treated with beta-blockers compared with controls. (see also weight values on the right).

**Figure 3:**
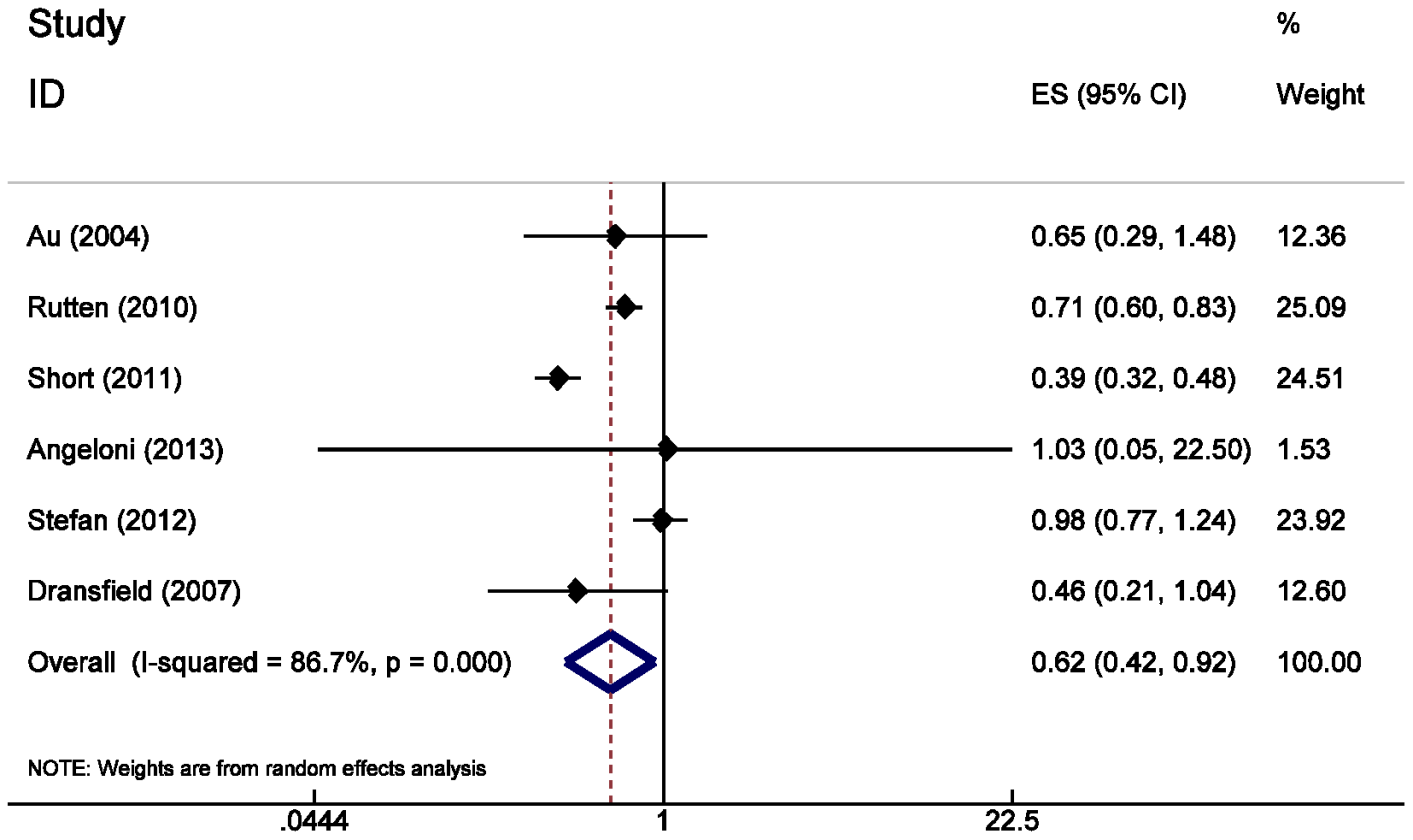
Forest plot of beta-blockers use and exacerbation of COPD risk in COPD patients.

Treatment with beta-blockers was associated with a significantly decreased risk for mortality (RR 0.72; 95% CI = 0.63 to 0.83) (Fig. 2) and exacerbation (RR 0.63; 95% CI = 0.57 to 0.71) (Fig. 3) in COPD patients. These results were highly significant (p = 0.00001). The risk for mortality was more significantly decreased in COPD patient with coronary artery disease (RR 0.64; 95% CI = 0.54 to 0.76) and heart failure (RR 0.74; 95% CI = 0.58 to 0.93).

### Interstudy Variability

**Figure 4:**
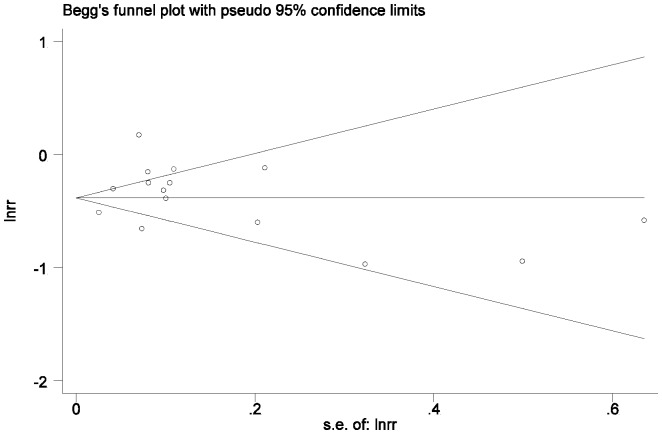
Sensitivity analysis of the meta-analysis of the association between beta-blockers use and mortality risk in COPD patients. The meta-analysis is dominated by the Gottlieb study and Ekstrom study.

The pooled relative risk of COPD related mortality secondary to beta-blockers use was 0.72(95% CI: 0.63–0.83; I_2_ = 89%, p = 0.00001). Our results indicate a high degree of heterogeneity among the included studies (Fig. 2). In subgroup analysis, no evidence of heterogeneity was noted in the analysis of mortality RR in the COPD patients with heart failure, with a p value for heterogeneity of 0.209. We used the leave-one-out sensitivity analyses by removing one study per time to check if individual study influenced the results. In the sensitivity analysis, we identified 2 studies [6],[37] as the source of heterogeneity (Fig. 4). Exclusion of these 2 studies from the analysis removed the study heterogeneity in the subgroup with nonspecific disease (I^2^ = 5.7%, p = 0.38), and only removed a little heterogeneity in the subgroup of COPD patients with coronary artery disease, while the pooled RR stayed significantly protective (RR = 0.74, 95% CI, 0.70–0.79).

### Publication Bias

**Figure 5:**
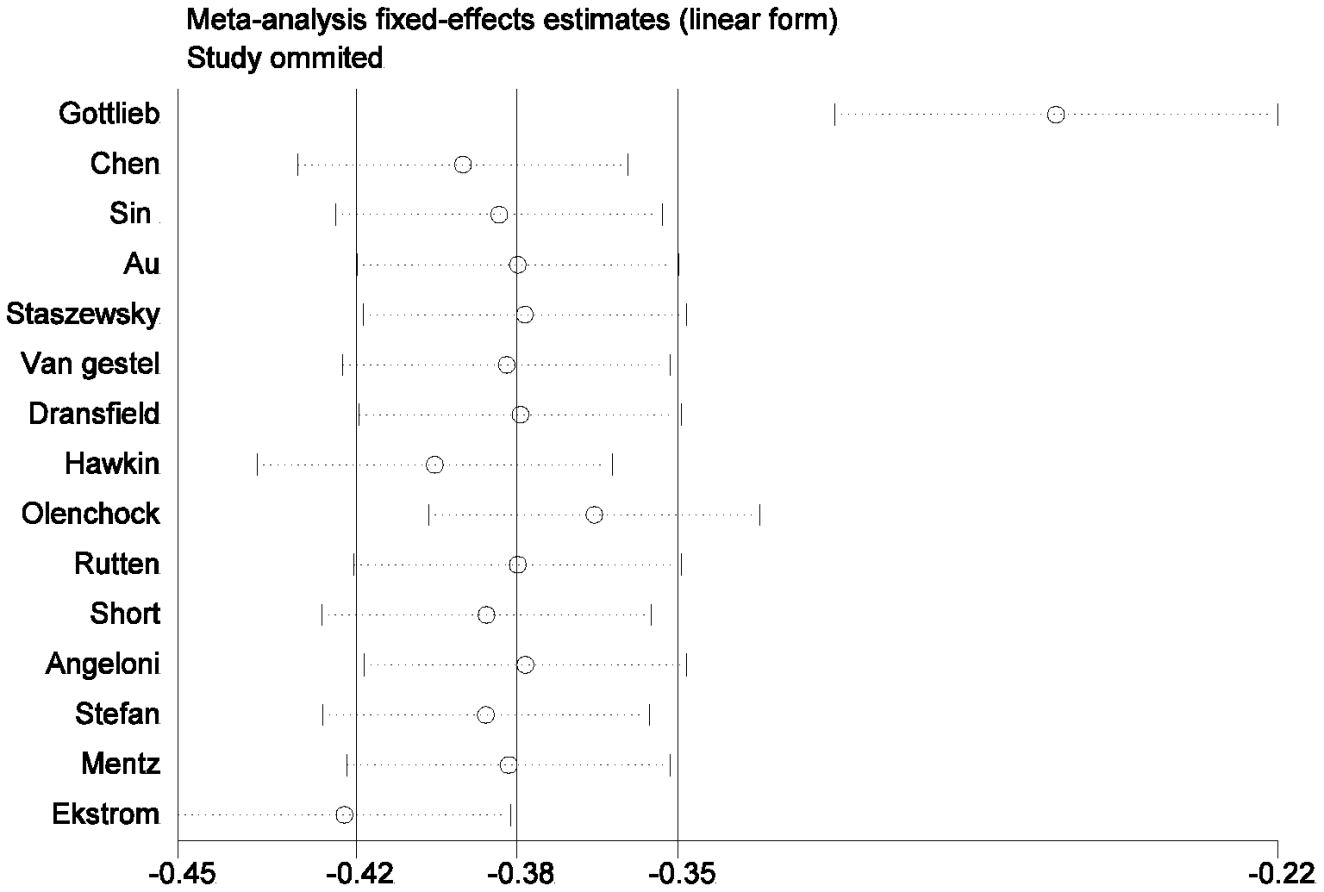
Begg’s Funnel Plots with Pseudo 95% Confidence Limits for studies reporting beta-blockers use and mortality in COPD patients. There is no evidence of bias in the test or the formal plot (t = 0.90, p = 0.382).

We used Egger’s regression asymmetry test to access the publication bias of literatures and no publication bias was found (t = 0.90, p = 0.382) (Fig. 5).

## Discussion

In comparison with the well-informed benefits of beta-blockers in patients with cardiovascular diseases, the effect of beta-blockers in patients with COPD is uncertain. The competing benefits and risks of beta-blockers use in patients with obstructive airway diseases have been a topic of debate. Beta-blockers are the mainstay of therapy for cardiac diseases, with studies demonstrating sustained improvements in survival and reduced morbidity [41]–[43]. Evidence that beta-blockers use in patients with obstructive airway diseases is associated with a reduction in morbidity and mortality also has been accumulating over the past 20 years. More recently, evidence indicates that the majority of patients with obstructive airway diseases can safely tolerate beta-blockers therapy, and it may lead to a reduction in respiratory exacerbation and death.

This meta-analysis pooled cohort studies on the use of beta-blockers in patients with COPD, and demonstrated that beta-blockers produced significant reduction in mortality and exacerbation. The findings of this analysis are supported by two other meta-analyses. One demonstrated that cardioselective beta-blockers given to patients with reversible airway disease increased response to beta-agonists and did not produce clinically significant adverse respiratory effects [19], and the other demonstrated that beta-blockers were associated with a lower relative risk of mortality 0.69 (95% CI: 0.62–0.78), but the latter only included 9 studies and indicated the presence of bias [38]. Neither of them assessed the effect of beta-blockers on the exacerbation of COPD. Here we included 6 more recent studies and the examination of the funnel plot revealed no presence of publication bias.

This meta-analysis reveals that beta-blockers have a significantly protective effect on mortality in COPD patients. The sensitivity analysis showed that the mortality benefit of beta-blockers in this analysis was largely driven by the results of Gottlieb [23] and Ekström [37]. This may be due to the fact that rate ratio in the first study was unadjusted for any confounder, and the latter study was a prospective cohort study and looked at subjects with severe COPD. However, the protective effect still remained after removing these studies. The conclusion that the treatment of beta-blockers reduces mortality in patients with COPD is fairly reliable.

The potential mechanisms underlying the benefits of beta-blockers treatment on all-cause mortality and respiratory event in patients with COPD have not been clarified. In this meta-analysis the mortality benefits were more significant in subgroup analyses of COPD patients with heart failure or coronary heart disease. It might be due to the anti-hypertension effect, arrhythmic-risk reduction and myocardial perfusion improvement by beta-blockers. It has been found that the activation of sympathetic nervous system in patients with COPD is increased. The increased sympathetic activation is associated with increased heart and respiratory rates, and ventilatory dead space [45]. It is also associated with decreased exercise-induced vasodilatation in skeletal muscles, loss of muscle fibers, and impaired endothelial function [27]. Beta-blockers are known to temper the sympathetic activation, including heart rate reduction. Clinical studies have shown that heart rate is an independent factor for all-cause mortality in individuals with or without cardiac disease [46],[47]. It has been proven that most patients with COPD and coronary heart disease present inadequate heart rate control and frequent angina episodes, and underuse of beta-blockers therapy largely contributes to the inadequate heart rate control [18]. Beta-blockers have been shown to reduce total mortality and sudden cardiac death by reducing heart rate and prolonging the diastolic period of cardiac cycle, thus improving myocardial perfusion [4].

The beneficial effect of beta-blocker use on the exacerbation in COPD may be of particular importance, as the prevention of acute exacerbation is one of the goals in the long-term management of COPD. It is tempting to associate this effect with improvements in cardiovascular diseases, resulting in reduced shortness of breath, a cardinal symptom of COPD exacerbation. However, this effect still remained after adjusting for cardiovascular diseases, indicating that beta-blockers have an independent effect on COPD itself. In animal studies, chronic treatment with beta-blockers was accompanied by a protective effect on airway responsiveness to methacholine in sensitized and challenged mice [39]. An experimental study showed that beta-blockers not only reduced the inflammatory cells in the bronchoalveolar lavage of antigen challenged mice, but also reduced the levels of cytokines, such as IL-13, IL-10, IL-5, and TGF-β_1_ [40]. In addition, chronic treatment with beta-blockers also produced a marked time-dependent decrease in goblet cells and mucin content of the airway epithelium [40]. It is shown in animal models that beta-blockers up-regulate beta2-receptors in the lung and thus improve the effectiveness of bronchodilators [48]. A study in humans supports this finding by showing that chronically titrating doses of beta-blockers in asthma patients reduced airway hyperresponsiveness [44]. Taken together, chronic beta-blocker use in COPD may produce beneficial results through anti-inflammatory and bronchoprotective effects. Long-term trials are needed to assess the safety and efficacy of beta-blockers in patients with COPD and concomitant cardiovascular diseases. Furthermore, evidence is also needed to clarify whether beta-blockers are beneficial to COPD patients without cardiovascular comorbidities.

## Study Limitations

This meta-analysis has several limitations, some similar to those found with most meta-analysis and therefore making it difficult to reach definitive conclusions. There is a marked heterogeneity noted in study size, duration, and the mean ages of the patients. There is also significant heterogeneity in phenotypes of the concomitant diseases with COPD and COPD itself. The analysis only reports on published literature and is therefore subject to publication bias, although funnel plots of effect size versus standard error for the studies in this review showed no evidence of bias. Furthermore, most of the effect estimates [24]–[26],[28],[29],[32],[33],[35],[37] are adjusted for confounders, but the level of adjustment is different between studies (Table 1), and this heterogeneity might have an effect on the outcome. Finally, the available data are limited for a powerful meta-analysis of the beta-blockers’ effect on outcomes of functional status and symptoms.

Another limitation of our study is that most studies are nonrandomized and retrospective in design and are subject to some confounders. Firstly, the diagnosis of COPD relied on physician diagnosis rather than strict clinical criteria, and most of the studies didn’t provide pulmonary function or severity of COPD. It is possible that patients treated with beta-blockers may have less severe baseline COPD or heart failure. We were unable to stratify our analysis on the presence, phenotype, or severity of COPD and heart failure, since these data were not available. Secondly, adherence to the prescribed beta-blockers is an imperfect assumption, so immortal time bias and calendar time bias are inevitable in these studies, as mentioned in Etminan’s study [38]. Finally, only three studies [33],[35],[36] provided type of the beta-blockers and none of the studies in this meta-analysis provided the specific doses administrated. Therefore we were unable to assess the benefit of the type and doses of beta-blockers on the risk of mortality and exacerbation of COPD. Despite these limitations, we believe that this analysis should add more evidence to the respiratory and mortality benefit of beta-blocker use in patients with COPD.

## Conclusions

This analysis reinforces the accumulating evidence that beta-blocker use is safe in patients with obstructive airway disease and is associated with significant reduction in mortality and exacerbation of COPD. Our analysis estimates the effect benefit of beta-blockers on mortality of obstructive lung diseases, and this effect is likely to be greater in patients with pre-existing heart disease. Considering the benefits of beta-blockers in conditions like hypertension, heart failure and coronary artery disease, these drugs should not be withheld because of COPD.

## Supporting Information

Table S1 The Quality of Cohort Studies Assessed by Newcastle-Ottawa Scale.
(DOCX)

Checklist S1 PRISMA Checklist.
(DOC)

Material S1 Includes: 1. Primary data for figure 2 to 5; 2. Command for figure 2 to 5.
(RAR)
