# Supplementary material for: Beta-Blockers Reduced the Risk of Mortality and Exacerbation in Patients with COPD: A Meta-Analysis of Observational Studies
Source: PLoS One. 2014 Nov 26;9(11):e113048. doi: 10.1371/journal.pone.0113048 (PMC4245088; doi:10.1371/journal.pone.0113048)
Supplement: Table S1 — The Quality of Cohort Studies Assessed by Newcastle-Ottawa Scale. (DOCX) [file pone.0113048.s001.docx]

**Table S1: The Quality of Observational Studies Assessed by Newcastle – Ottawa Scale**

|  |  | **Newcastle –Ottawa Scale** | | | | | | | | | |
| --- | --- | --- | --- | --- | --- | --- | --- | --- | --- | --- | --- |
|  |  | **Selection** | | | | **Comparability** | | **Outcome** | | | **Total stars** |
| **Study** | **Year** | **1** | **2** | **3** | **4** | **5A** | **5B** | **6** | **7** | **8** |  |
| Gottlieb/ [20] | 1998 | A(*) | A(*) | A(*) | A(*) | A(*) | No | B(*) | A(*) | B(*) | 8 |
| Chen/ [21] | 2001 | A(*) | A(*) | A(*) | A(*) | A(*) | B(*) | B(*) | A(*) | B(*) | 9 |
| Sin/ [22] | 2002 | B(*) | A(*) | A(*) | B(0) | A(*) | B(*) | B(*) | A(*) | B(*) | 8 |
| Staszstaszewsky/ [23] | 2007 | A(*) | A(*) | A(*) | B(*) | A(*) | B(*) | B(*) | A(*) | B(*) | 9 |
| Au/ [24] | 2004 | A(*) | A(*) | A(*) | A(*) | A(*) | B(*) | B(*) | A(*) | A(*) | 9 |
| Dransfield/ [25] | 2007 | A(*) | A(*) | A(*) | A(*) | A(*) | No | B(*) | A(*) | A(*) | 8 |
| Van Gestel/ [26] | 2008 | B(*) | A(*) | A(*) | A(*) | A(*) | No | B(*) | A(*) | A(*) | 8 |
| Hawkins/ [27] | 2009 | A(*) | A(*) | A(*) | A(*) | A(*) | No | B(*) | A(*) | A(*) | 8 |
| Olenchock/ [28] | 2009 | A(*) | A(*) | A(*) | A(*) | A(*) | No | B(*) | A(*) | A(*) | 8 |
| Rutten/ [29] | 2010 | A(*) | A(*) | A(*) | A(*) | A(*) | No | B(*) | A(*) | A(*) | 8 |
| Short/2011[30] | 2011 | A(*) | A(*) | A(*) | A(*) | A(*) | B(*) | B(*) | A(*) | A(*) | 9 |
| Stefan/ [31] | 2012 | A(*) | A(*) | A(*) | A(*) | A(*) | B(*) | B(*) | A(*) | B(*) | 9 |
| Ekström/ [32] | 2013 | A(*) | A(*) | A((*) | A((*) | A(*) | No | B(*) | A(*) | A(*) | 8 |
| Angeloni/ [33] | 2013 | A(*) | A(*) | A(*) | A(*) | A(*) | B(*) | B(*) | A(*) | A(*) | 9 |
| Mentz/ [34] | 2013 | A(*) | A(*) | A(*) | A(*) | A(*) | B(*) | B(*) | A(*) | B(*) | 9 |
